# Age-related changes to the neural correlates of working memory which emerge after midlife

**DOI:** 10.3389/fnagi.2014.00070

**Published:** 2014-04-16

**Authors:** Helen N. Macpherson, David J. White, Kathryn A. Ellis, Con Stough, David Camfield, Richard Silberstein, Andrew Pipingas

**Affiliations:** ^1^Centre for Human Psychopharmacology, Swinburne UniversityHawthorn, VIC, Australia; ^2^Department of Psychiatry, Academic Unit for Psychiatry of Old Age, St. Vincent’s Aged Psychiatry Service, St. Georges Hospital, University of MelbourneMelbourne, VIC, Australia

**Keywords:** working memory, SSVEP, aging, midlife, middle age, compensation

## Abstract

Previous research has indicated that the neural processes which underlie working memory change with age. Both age-related increases and decreases to cortical activity have been reported. This study investigated which stages of working memory are most vulnerable to age-related changes after midlife. To do this we examined age-differences in the 13 Hz steady state visually evoked potential (SSVEP) associated with a spatial working memory delayed response task. Participants were 130 healthy adults separated into a midlife (40–60 years) and an older group (61–82 years). Relative to the midlife group, older adults demonstrated greater bilateral frontal activity during encoding and this pattern of activity was related to better working memory performance. In contrast, evidence of age-related under activation was identified over left frontal regions during retrieval. Findings from this study suggest that after midlife, under-activation of frontal regions during retrieval contributes to age-related decline in working memory performance.

## Introduction

Working memory is vulnerable to the effects of age and changes to the neural correlates of working memory occur across the lifespan (Babcock and Salthouse, 1990; Rypma and D’Esposito, 2000). Some investigations have demonstrated that there are age-related changes in the activity of the dorsolateral region of the prefrontal cortex (PFC; Rypma and D’Esposito, 2000; Rypma et al., 2001). Other studies examining working memory have revealed that the parietal and occipital neural processes which direct sensory processing and attention (Zimmer, 2008) are attenuated in the elderly, and these posterior reductions are coupled with a greater recruitment of frontal neural resources (McEvoy et al., 1998; Müller and Knight, 2002).

Increased reliance on frontally-mediated executive processes may represent a compensatory mechanism which is beneficial for cognitive performance (Reuter-Lorenz and Cappell, 2008), indicating that not all age-related differences in brain activation represent declining cognitive performance. Functional reorganization of the PFC in the form of increased bilateral activity has been identified in studies of working memory (Reuter-Lorenz et al., 2000; Mattay et al., 2006) and has been demonstrated to be conducive to successful task performance (Cabeza et al., 2002). To be considered compensatory, increased cortical activity in seniors must be deemed to be beneficial to cognitive performance. Additional activity must be observed in high performing seniors or when performance is equivalent to younger adults. Based on this definition, it is recognized that age-related increases in activation can represent both compensatory processes in some brain regions and concomitant loss of neural specialization in others (Rossi et al., 2004; Davis et al., 2008). It has also been postulated that such compensatory processes may reflect the adoption of reactive rather than proactive control strategies (Paxton et al., 2008), leading to age-equivalent performance in the presence of differential patterns of neural activation (Grady, 2012).

Whilst PET and fMRI techniques have provided valuable insights regarding neurocognitive aging, the temporal resolution of these regional metabolic measures do not approach the millisecond accuracy of event related potential (ERP) brain electrical techniques (de Haan and Thomas, 2002). The steady state visually evoked potential (SSVEP) is a measure of brain electrical activity which possesses the unique ability to monitor brain activity associated with ongoing processing demands (Silberstein, 1995). The SSVEP is capable of delineating working memory sub-processes of encoding, maintenance and retrieval (Silberstein et al., 2001; Ellis et al., 2006; Macpherson et al., 2012) and can provide insights into excitatory and inhibitory processes in the brain (Silberstein et al., 2000b; Kemp et al., 2004). The SSVEP consists of amplitude and phase. The amplitude represents the magnitude of the SSVEP, whilst the phase is the difference between the visual sinusoidal stimulus and the SSVEP. Phase is expressed as latency to provide a measure of transmission speed through the brain. Alterations in 13 Hz SSVEP activity have been suggested to reflect activity within cortical pyramidal cells (Kemp et al., 2004). A reduction of the SSVEP latency has been interpreted as increased post-synaptic excitation of these pyramidal neurons and latency increase to reflect an increase in post-synaptic inhibition (Silberstein et al., 2000a). The specific paradigm utilized to record the SSVEP is known as steady state topography (SST), a technique in which the SSVEP is elicited by a 13 Hz uniform flicker, delivered via light emitting diode (LED) goggles, and superimposed on any visual cognitive task.

SST studies in younger adults have revealed that during working memory encoding, SSVEP amplitude is reduced at left parietal regions (Silberstein et al., 2001) and across bilateral frontal sites (Ellis et al., 2006). Due to the proximity of 13 Hz activity to the α-band, 13 Hz SSVEP amplitude reductions during memory encoding are thought to be closely related to attentional processes (Silberstein et al., 1990) and have been equated to event related desynchronization (ERD) in the upper α-band (Silberstein, 1995). ERD refers to a decrease of power in the EEG and the magnitude of ERD corresponds to the extent of regional cortical activation (Klimesch et al., 2006). SSVEP latency reductions have been interpreted as increased neural excitation and, when present with amplitude reductions, are indicative of increased cortical activity (Silberstein, 1995; Kemp et al., 2004). As opposed to encoding, the dominant feature of the maintenance period has been increased SSVEP amplitude at occipital and prefrontal sites (Silberstein et al., 2001; Perlstein et al., 2003; Wu and Yao, 2007). The functional significance of this amplitude increase has been considered to reflect increases in the transmission efficiency of the cortico-cortical re-entrant loops associated with holding information “on-line” (Silberstein et al., 2001).

Previously we have identified age differences in the SSVEP during memory activation (Macpherson et al., 2009). In this study we investigated age differences in the performance of a low demand object working memory delayed response task and a more difficult contextual recognition task in older (59–67 years) and younger (20–30 years) adults. During working memory maintenance, younger adults demonstrated greater frontal SSVEP amplitude and latency reductions than older adults. As this is indicative of reduced cortical activation in the older adults, relative to the younger group, this age difference in activity was interpreted as an age-related decrease in neural processes. In particular, the characteristic working memory-related SSVEP amplitude increases previously observed in other studies (Silberstein et al., 2001; Perlstein et al., 2003) were attenuated in the elderly. In contrast, during the memory retrieval component of the more difficult contextual recognition task, older adults demonstrated larger, more extensive, posterior SSVEP amplitude and latency reductions, indicative of age-associated compensatory processes.

Generally, studies of neurocognitive aging have compared brain activity of a cohort of young adults to that of an older group. A potential limitation of these trials is that brain maturation continues into the fourth decade, thus some of the age-related changes in neural function may represent development in the younger group (Aine et al., 2006, 2011). Lifespan studies have indicated changes in memory task-related neural activity may emerge from middle age onward (Grady et al., 2006; Mattay et al., 2006; Park et al., 2013). However few studies have examined which components of working memory show the greatest age-related changes in neural activity, with respect to a middle-aged comparison group.

The 13 Hz SSVEP provides a continuous measure of time-variable cognitive processes enabling the examination of neural activity with high temporal resolution across all stages of a working memory task. In the current investigation we extend on our previous work (Macpherson et al., 2009) to examine age differences in the neural processes which underlie working memory encoding, maintenance and retrieval. In this study we focus on age differences which occur after midlife in a large sample of 130 individuals. Our prior study identified age-associated increases in neural activation during episodic retrieval, but not the maintenance period of a working memory task (Macpherson et al., 2009). Others have tended to indicate that age-related compensatory patterns of neural activity may occur during memory encoding (Craik and Rose, 2012). On the basis of these findings, we would anticipate that age-related increases in cortical activation (i.e., SSVEP amplitude and latency reductions), indicative of compensatory processes, would occur during working memory encoding and possibly retrieval, but not maintenance.

## Materials and methods

### Participant demographics

SSVEP data was obtained from a total of 141 individuals and data from 130 participants was included in this study. Participants were aged between 40 and 82 years (M = 60.4 years, SD = 10 years), right-handed, non-smokers, with no history of stroke, epilepsy, dementia, Parkinson’s disease, head trauma, excessive alcohol use, mental illness, depression, anxiety disorders and were not using anti-depressant, anti-anxiety medication or any medications with a cognitive enhancing effect. Participants aged over 65 were required to have a mini mental status exam (MMSE) score above 25 in order to participate. Participants provided informed consent and ethics approval was provided by the Swinburne University Human Research Ethics Committee. SSVEP data obtained from 11 participants was excluded from analysis due to EEG processing errors. Participants were divided into a middle aged (40–60 years, *n* = 66) and older group (61–82 years, *n* = 64). Participant demographics are shown in Table 1. Older participants had completed significantly less education than their midlife counterparts (*t*_(126)_ = 3.74, *p* < 0.001).

**Table 1:** **Participant demographics**.

**Characteristic**	**Middle age 40-60 years**	**Older 61-82 years**
	**Mean (SD)**	**Mean (SD)**
Age	52 (6)	69 (5)
Gender (men/women)	30/36	18/46
Years Education	16 (3)	14 (4)

### Procedure

During the brain activity recording session participants were seated approximately 1.3 m from the task computer and fitted with half-silvered mirror goggles. Cognitive task stimuli were presented on a computer with a 14 inch liquid crystal display (LCD) monitor. Brain electrical activity was recorded whilst participants completed a spatial working memory task and a control task. Both tasks were preceded by practice trials to reduce novelty effects and to ensure participants were comfortable with the task requirements.

### EEG recording and SSVEP stimulus properties

EEG was recorded from 64 tin, scalp electrodes, embedded in a lycra cap. Linked earlobes were used for reference and the nose was used for the ground. Impedance was below 10 kΩ for reference electrodes. Brain electrical activity was amplified and band-pass filtered 3 dB down at 0.1 and 80 Hz prior to digitization to 16-bit accuracy at a rate of 500 Hz. Participants were fitted with goggles comprised of two sets of LED arrays viewed through half-silvered mirrors. The goggles were used to superimpose a flickering white light on the participant’s visual field. The SSVEP was evoked by a 13 Hz sinusoidal flicker subtending a horizontal angle of 160° and a vertical angle of 90°. The visual flicker covered the majority of the visual field with a modulation depth of the stimulus against the background of 45%.

### Spatial working memory task

**Figure 1:**
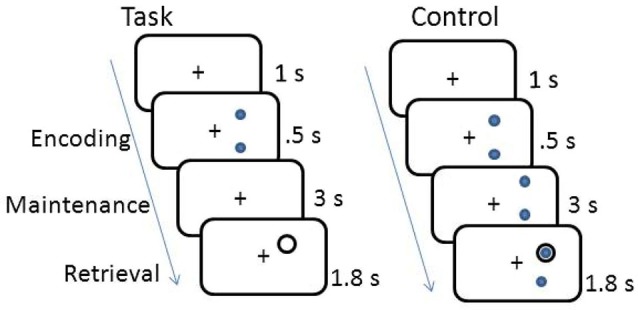
**Schematic of working memory and control tasks**.

The working memory task was a delayed response task. The working memory and control tasks are shown in Figure 1. Participants memorized the location of either 2 or 3 dots displayed on the circumference of an imaginary circle for 0.5 s, and then retained the location of the dots in memory across a 3 s fixation interval. A probe circle was presented for 1.8 s and participants determined whether the location of the probe circle matched the location of one of the stimulus dots shown during encoding. Each trial was separated by a 1 s fixation period. Subjects responded with a right hand button press if the probe circle location matched the stimulus dot location and a left hand button press if it was a non-match. The control task was the same, except the dots remained on the screen for the entire duration of the trial. Participants completed two blocks of 40 trials of the spatial working memory task and one block of 40 trials of the control task.

### SSVEP signal processing

SSVEP signal processing was conducted using BrainSci (SSPT Analysis Software, version 2). To examine the SSVEP associated with the working memory task, changes to the SSVEP were determined from the 13 Hz Fourier coefficients evaluated over 10 stimulus cycles at the 13 Hz stimulus frequency, resulting in a temporal resolution of 0.77 s. The 10 cycle evaluation period was then shifted one stimulus cycle and the Fourier coefficients recalculated for this period. This process was applied to all 64 scalp electrode sites. 6.3 s epochs of data, centered on the presentation of the fixation cross and associated with correct task responses, were extracted from the Fourier time series and averaged. SSVEP amplitude and latency were normalized. Specifically, the mean amplitude was calculated for each electrode for the duration of the 6.3 s control task epoch. The values for each electrode were then averaged to create a normalization factor for each individual and amplitude values were divided by the subject’s unique normalization factor. SSVEP phase was normalized by subtracting the average phase for each electrode from the phase of the corresponding electrode site. Phase is expressed as latency to provide a measure of transmission speed through the brain. Further details of SSVEP analysis (Silberstein, 1995) and applications (Vialatte et al., 2010) are described elsewhere.

### Statistical analysis

#### Behavioral data

Behavioral data was analyzed using SPSS software version 20 (IBM Corp©, USA). Analysis of covariance (ANCOVA) was used to control for the effects of gender, due to gender imbalance across the age groups. Between groups repeated measures ANCOVA was used to compare age effects in relation to response time and accuracy of the working memory task and the control task, whilst controlling for the effects of gender. A performance composite score was created by dividing the accuracy of the working memory task by response time. One way ANCOVA was used to compare the composite performance score of the middle aged group to the older group, whilst controlling for gender.

#### SSVEP data

SSVEP data processing was carried out using BrainSci (SSPT Analysis Software, version 2) and statistical comparisons and mapping were conducted in MATLAB version 7.12 (R2011a, The Mathworks). Hotelling’s *T*-tests were used to estimate the probability of falsely rejecting the null hypothesis (type-1 error), associated with task differences in the SSVEP latency and amplitude. Hotelling’s *T* is the multivariate counterpart of Student’s *T*-test and is used to account for both the amplitude and latency components of the SSVEP.

### Working memory task effects across all participants

A within groups Hotelling’s *T*-test was used to compare the SSVEP amplitude and latency time series associated with the spatial working memory task to that of the control.

### Working memory age differences

Pearson’s *r* correlations were calculated to examine the relationship between age and SSVEP amplitude and latency across the entire working memory task. To account for to the large number of multiple comparisons, correlations were only considered to be significant if they were observed at a minimum of three successive time points and three electrodes within the same region. Between groups Hoteling’s *T*-tests were used to compare the SSVEP amplitude and latency associated with the spatial working memory task for the older group compared to the midlife group.

## Results

### Behavioral results

**Figure 2:**
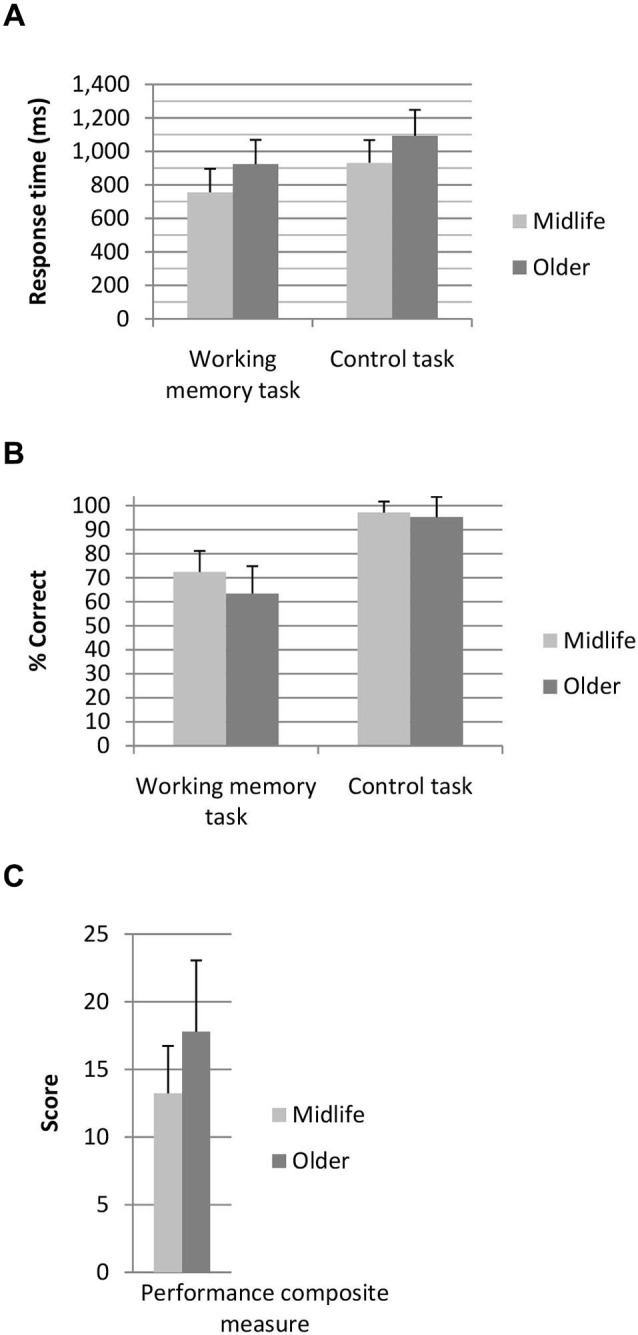
**(A)** Response time and **(B)** percentage correct for the working memory and control tasks and **(C)** combined performance score for the working memory task. Performance composite score, where lower score indicates superior working memory performance.

Participants performed the 3 dot trials at a higher level of accuracy than the 2 dot trials (*t*_(128)_ = −5.03, *p* < 0.001), however the 3 dot trials were performed significantly slower than the 2 dot trials (*t*_(128)_ = −3.70, *p* < 0.001). As clear grading effects were not elicited by the task (i.e., accuracy and response time differences between the 2 and 3 dot conditions were not in the same direction) 2 and 3 dot trials were combined for all further analysis. A breakdown of performance across the two age groups for the working memory task and the control task are shown in Figure 2. A main effect of task (*F*_(1,127)_ = 55.44, *p* < 0.001, *η*^2^ = 0.31), age group (*F*_(1,127)_ = 15.68, *p* < 0.001, *η*^2^ = 0.11) and a task by age interaction (*F*_(1,127)_ = 16.80, *p* < 0.001, *η*^2^ = 0.12) were identified for task accuracy. Both age groups performed the control task at a higher level of accuracy than the working memory task, whilst older adults performed the working memory task at a lower level of accuracy than the midlife group. In terms of response time a main effect of task (*F*_(1,127)_ = 17.13, *p* < 0.001, *η*^2^ = 0.12) and age group (*F*_(1,127)_ = 44.52, *p* < 0.001, *η*^2^ = 0.26) were identified. Response time on the control task was faster for the working memory task and older adults performed slower than the midlife group on both tasks. Older adults performed significantly poorer than the midlife group on the combined working memory composite measure (*F*_(1,127)_ = 29.16), *p* < 0.001, *η*^2^ = 0.19).

### SSVEP results: working memory task effects across all participants

**Figure 3:**
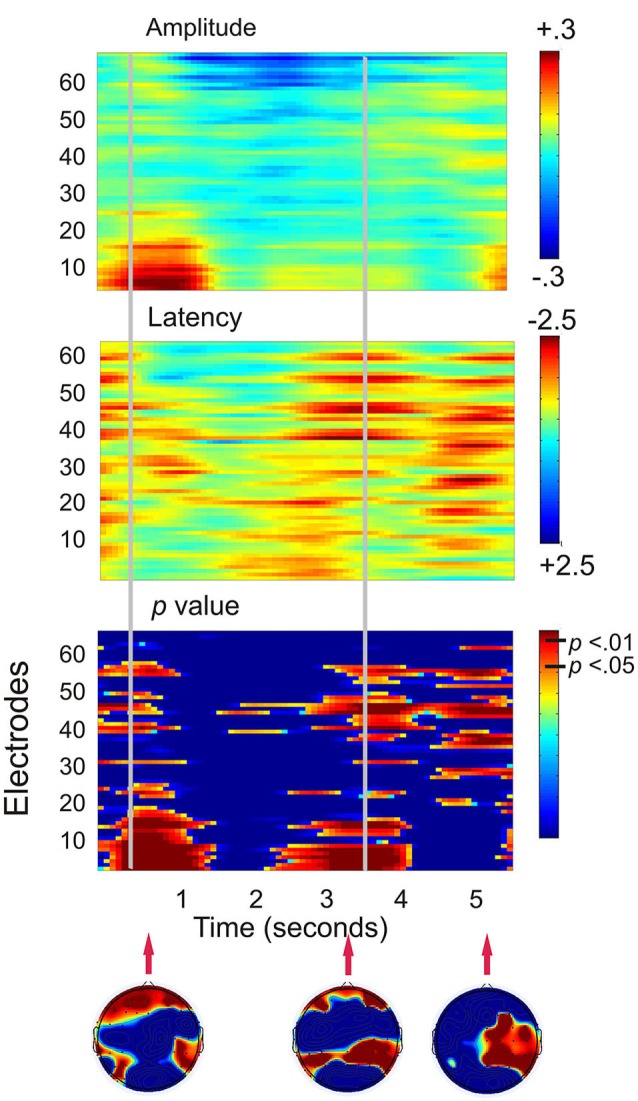
**Plots displaying the working memory task effects for all 130 participants (SSVEP amplitude and latency for the working memory task compared to the control task)**. Time is presented on the *x*-axis and electrodes on the *y*-axis. Vertical event marker lines separate encoding, maintenance and retrieval stages of the task. Warmer colors indicate amplitude decreases and latency decreases for the spatial working memory task relative to the control task. Cooler colors indicate amplitude and latency increases. Hotelling’s *T* statistical significance is shown below, topographical maps display maximal statistical effects.

Figure 3 shows that when the working memory task was compared to the control task, the largest statistical effects (*p* < 0.01) occurred in two major frontal profiles of activity. The first cluster was predominantly associated with a bilateral prefrontal and frontal amplitude reduction from encoding to the first 800 ms of the maintenance period. The second profile of activity was a bilateral prefrontal and temporal-parietal latency reduction beginning at the second half of the maintenance period and continuing into retrieval until approximately 300 ms following the presentation of the probe. A final more posterior pattern of activity was a latency reduction across right temporal-parietal to midline sites including Cz and PZ, at a time corresponding to the average participant response time.

### SSVEP results: working memory age differences

**Figure 4:**
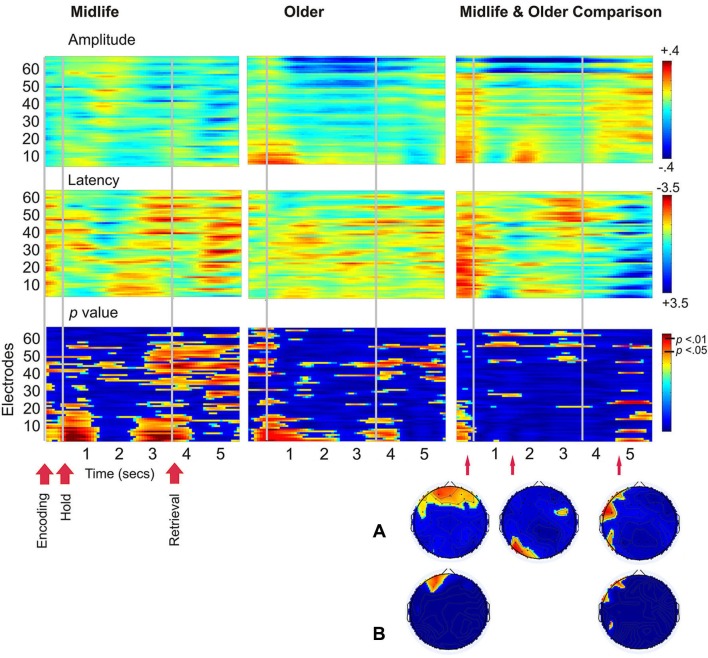
**Left and central plots display SSVEP amplitude and latency for the working memory task compared to the control task separately for the midlife and older groups**. *p*-value plots below indicate within group statistical effects for the working memory task compared to the control task. Plots on the far right display the direct comparison of the working memory task in the older adults to the midlife group. Warmer colors indicate amplitude decreases and latency decreases for older adults relative to early midlife. The Hotelling’s *T* *p*-value plot below indicates the between groups statistical effects for the working memory task in the older group compared to the midlife group. **(A)** Topographical maps display maximal statistical effect for the older group compared to the midlife group. **(B)** Topographical maps display maximal statistical effect for a subset of the older group compared to a subset of the midlife group, when the sample is split into tertiles to compare the oldest (67–82 years) to the youngest (40–56) participants.

There were no correlations between age and the SSVEP which met the criteria for statistical significance, therefore a figure of these results is not shown. Figure 4 demonstrates age differences in SSVEP amplitude, latency and the topography of the maximal statistical effects for each stage of the working memory task. Age differences were apparent early into the encoding period with older adults demonstrating greater bilateral prefrontal and frontal SSVEP amplitude and latency reductions (*p* < 0.05). Older adults displayed an increase in SSVEP latency at left parietal occipital sites across the entire task and this pattern of activity reached statistical significance 0.9 s into the 3 s maintenance period. During retrieval older adults demonstrated significantly smaller SSVEP amplitude reductions across left prefrontal, frontal and temporal sites when compared to the midlife group.

### Correlation between SSVEP and performance

**Figure 5:**
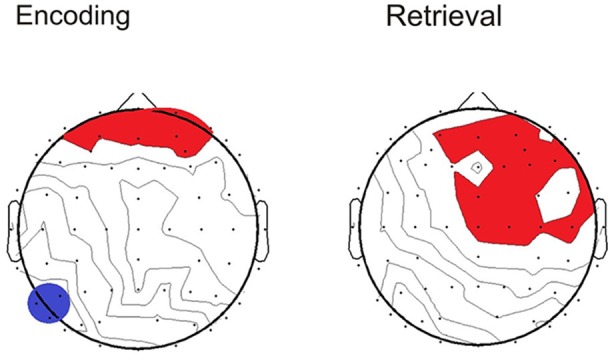
**Topographical maps display the Pearson’s *r* correlation between task performance and SSVEP amplitude for the older group**. Time points correspond to the topographical maps shown in Figure 4. Red highlighting indicates a positive relationship and blue indicates negative (*p* < 0.05).

**Figure 6:**
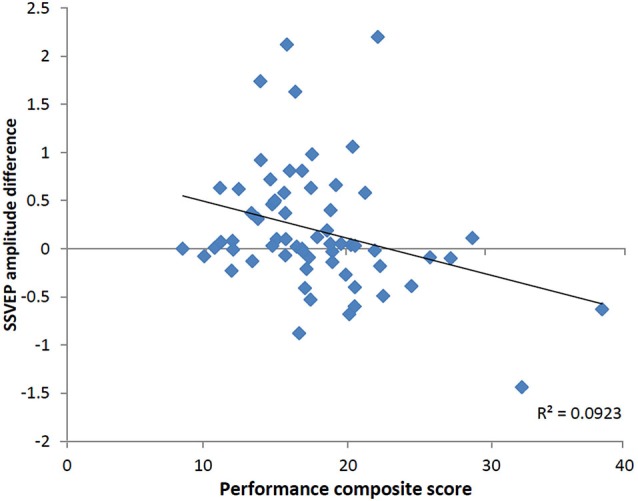
**Scatterplot displaying relationship between SSVEP amplitude reductions and the working memory performance score at right prefrontal electrode FP2 in the older group (Pearson’s *r* = −0.30, *p* < 0.05)**. Timing corresponds to the encoding time point shown in the correlation map in Figure 5. Positive values indicate larger SSVEP amplitude reductions for the working memory task relative to the control task and larger performance score is indicative of poorer task performance.

To determine whether the pattern of neural activity displayed by the older adults was beneficial to performance, SSVEP amplitude (working memory task and control task difference) was correlated with the performance composite measure for the same time points displayed in the topographical maps in Figure 4. As shown in Figure 5, during encoding, greater amplitude reductions across bilateral prefrontal regions were associated with better performance and the same pattern was shown across right prefrontal and central sites during retrieval (*p* < 0.05). SSVEP amplitude increases at left temporal-parietal sites were associated with better performance during encoding only (*p* < 0.05). SSVEP amplitude was not significantly related to performance during the relevant maintenance period. Figure 6 plots the correlation between SSVEP amplitude and performance during encoding.

## Discussion

Findings from this large study of middle aged and older adults demonstrate that age differences in cortical activation are not uniform across the encoding, maintenance and retrieval stages of a spatial working memory task. In line with current accounts of neurocognitive aging (Craik and Rose, 2012; Grady, 2012), both age-related increases and decreases in neural activity were identified. Older adults demonstrated greater bilateral frontal activity during encoding. Consistent with the premise of neural compensation, this pattern of activity was related to better working memory performance. This finding is in line with the observation that with advancing age, there is a functional reorganization of the PFC in the form of increased bilateral activity (Cabeza, 2002), and this phenomenon has been demonstrated to be conducive to successful task performance (Cabeza et al., 2002). In contrast, evidence of age-related under activation, rather than compensation, was identified over left frontal regions during working memory retrieval. Age-differences during working memory maintenance were minimal and did not predict task performance. Our results suggest that after midlife, deficits to retrieval contribute to age-related decline to working memory.

### Working memory encoding and retrieval

This study represents the largest SSVEP data set to investigate the neural correlates of working memory and therefore provides important insights into the spatio-temporal profile of each working memory stage. When examined across all 130 participants, there was a decrease in SSVEP amplitude and latency during encoding, largest over bilateral frontal regions. These amplitude decreases are similar to the pattern of activity displayed previously during working memory encoding in smaller samples (Silberstein et al., 2001; Ellis et al., 2006; Macpherson et al., 2012) and are comparable to ERD in the upper α-band (Silberstein, 1995). Analogous activity was also shown during retrieval, where α-ERD has been postulated to reflect memory search processes (Krause et al., 1996), potentially via the release of inhibitory processes which suppress access to memory (Klimesch et al., 2011), enabling the transient reactivation of long term memory (Ruchkin et al., 2003; Klimesch et al., 2006). Our findings of increased cortical activation during encoding and retrieval correspond to current theories proposed by Klimesch et al. (2011) and Klimesch (2012) who suggest that α-oscillations control access and retrieval of meaningful information and ERD reflects the release of inhibition which enables this to occur.

In terms of age differences during working memory encoding, there was an age associated increase in bilateral prefrontal cortical activation (i.e., larger SSVEP amplitude and latency reductions in older adults) observed during this stage of the task. According to the Hemispheric Asymmetry Reduction in Older Adults (HAROLD) model, some age-related increases in prefrontal neural activity may be described as compensatory (Cabeza et al., 2002), but this interpretation is dependent upon performance (Grady et al., 2005; Davis et al., 2008) and difficulty (Reuter-Lorenz and Cappell, 2008; Cappell et al., 2010) of the task. Few studies have examined whether compensatory neural processes can be detected with reference to a midlife group. During encoding, the older group in this study demonstrated larger SSVEP amplitude and latency reductions than the midlife group, over bilateral frontal regions. As this pattern of activity was related to better performance in the older group, this age-related increase in cortical activation is consistent with our definition of “compensatory” activity. We have previously shown SSVEP amplitude and latency reductions in both younger and older adults during the hold period of a low demand working memory task and the retrieval stage of a more difficult contextual recognition task (Macpherson et al., 2009). As amplitude and latency reductions were smaller in older adults than young adults during the easier task, and larger during the more complex memory task, we proposed that this pattern of activity represented an age-related compensatory recruitment of additional neural resources. In a comparison of the oldest (over 67 years) and youngest participants (under 56 years) in the current study, this pattern of activity was observed over left prefrontal regions only, and not across bilateral sites as shown in the larger sample. This finding suggest that the oldest adults in this sample may have reached their capacity limits (Reuter-Lorenz and Cappell, 2008) therefore resulting in the recruitment of fewer additional resources.

However, with lower performance identified in the older group, it has been suggested additional neural activity in older adults could also represent over-recruitment, which is not beneficial to task performance (Grady, 2012). It has been observed that SSVEP amplitude reductions increase with greater task difficulty (Ellis et al., 2006) in a similar manner to α-amplitude (Dujardin et al., 1993; Pfurtscheller and Lopes Da Silva, 1999) and upper α-ERD (Stipacek et al., 2003). The older group performed that task on average 9% less accurately than the midlife group. As such, the larger SSVEP amplitude and latency reductions demonstrated by the older adults during encoding could reflect the greater effort required to perform the task, or the recruitment of additional neural units which were not ultimately beneficial to performance (Logan et al., 2002; Zarahn et al., 2007). However, our finding that greater prefrontal activation during encoding was related to better performance amongst the older group does not support this interpretation.

If the age related increases in prefrontal and frontal SSVEP cortical activation identified in the current study do reflect compensatory processes which develop after midlife, it may be that the activity shown during encoding represents alterations to top-down, executive processes. An extensive body of electrophysiological and functional imaging literature supports the premise of an increased reliance by the elderly on frontally mediated executive processes (Grady et al., 1994; Rypma and D’Esposito, 2000; McEvoy et al., 2001; Reuter-Lorenz et al., 2001; Müller and Knight, 2002; Daselaar et al., 2003; Friedman, 2003; Davis et al., 2008; West et al., 2010). It has been proposed that this form of frontally-mediated compensatory neural activity may represent a different utilization of cognitive control (Grady, 2012). Top down control is the ability to adapt cognitive resources to continuously changing external demands and it has been suggested that older adults respond in a more reactive manner than younger individuals (Braver et al., 2007; Dew et al., 2012). This shift from proactive control to reactive control in aging has been attributed to declines to fluid intelligence and processing speed (Manard et al., 2014). It is possible that in the current study the increase in frontal activation during encoding could be due to alterations in strategy, although this cannot be confirmed due to the relatively simple working memory paradigm employed. Others have identified age-related differences in top-down control during working memory encoding in the form of a delay in the early stimulus specific processes (Zanto et al., 2010), as well as during the inhibitory top down suppression of task-irrelevant information at late selection processes (Werkle-Bergner et al., 2012).

Our results point to an age-associated decrease in frontal cortical activation during retrieval, with smaller SSVEP amplitude and latency reductions identified in older adults, relative to the midlife group. As correlations indicated that in the older group, similar to encoding, greater frontal activity was related to better performance, these findings suggest that a retrieval deficit may have contributed to the age-related decline in spatial working memory. This interpretation is consistent with previous data which has demonstrated that age differences were isolated to the retrieval phase of a working memory task, in the form of reduced retrieval-related neural activity in dorsolateral PFC (Rypma and D’Esposito, 2000). Age differences were significant in the second half of the retrieval period, emerging just prior to participant response. The timing and anterior location of this effect could equally implicate age-differences in the motor response, however older adults tend to demonstrate increased activation when initiating motor responses (Mattay et al., 2002; Riecker et al., 2006). Reduced activation of temporal-occipital and parietal regions has been identified during late recognition processes (Solesio-Jofre et al., 2012). In older adults temporo-occipital regions were under-recruited following retroactive interference in a face memory task. Reduced activity was interpreted by these researchers as an age-associated deficit to late top down processes due to inability to suppress irrelevant stimuli. It is plausible that in the current study, reduced frontal activity around the retrieval period in older adults may reflect increased vulnerability to interference, carried over from earlier trials. Alternatively, decreased retrieval- related activity could be due to failures to implement proactive strategies during memory encoding, however as strategy was not explicitly manipulated in the current paradigm this premise remains speculative.

### Working memory maintenance

During the maintenance period in the current study, the characteristic SSVEP amplitude increases associated with holding information in memory (Silberstein et al., 2001; Perlstein et al., 2003) did not reach statistical significance. Interestingly, this pattern of SSVEP amplitude increase during working memory maintenance has been most prominent when the task requires manipulation of memory content (Ellis et al., 2006) or a high level of demand (Silberstein et al., 2001), but appears attenuated during performance of single load delayed response tasks (Macpherson et al., 2009, 2012), such as the task used in the current study. These findings, along with the observations of the current study suggest that the requirement to recirculate information through the cortico-cortical re-entrant loops may depend on the complexity of the working memory operation.

Although SSVEP amplitude increases were not identified as the dominant feature of the maintenance period, older adults demonstrated larger parietal-occipital SSVEP amplitude increases than those in the midlife group. Interestingly, when the youngest and oldest participants from this sample were compared, this effect was no longer significant. In terms of a spatial working memory task, the parietal-occipital activation may reflect the greater effort of older adults to maintain the perceptual elements in memory. Alternatively, such age-associated changes to these regions may be indicative of a loss of posterior processing specificity, due to decreased neural specialization in the areas responsible for visual processing (Payer et al., 2006). This age effect appears to contradict our past findings which referenced a pre-midlife comparison group (Macpherson et al., 2009). The diverging result is most likely due to the overall greater difficulty of the spatial working memory delayed response task used in this study, than the object working memory task utilized in our previous investigation. Interestingly in this study, older adult’s performance was not related to the activity during the working memory maintenance period, and the greatest age differences in activation were observed during encoding and retrieval.

### Working memory encoding-maintenance distinction

In terms of the temporal dynamics of spatial working memory, the frontal and parietal-temporal SSVEP profile associated with the encoding and maintenance stages of the task was less differentiated in this large sample of middle aged and older adults, than previously observed in young adults (Silberstein et al., 2001; Ellis et al., 2006).

During working memory encoding, activation of the frontal and parietal regions are thought to reflect the control of selective attention and therefore the amount of information which can be encoded into memory (Gazzaley and D’Esposito, 2007). A recent review of imaging studies has suggested that this form of attention directing, top-down modulation continues to operate during working memory maintenance, and possibly retrieval as well (Gazzaley and Nobre, 2012). This premise has been strengthened by Todd et al. (2011) who used time resolved fMRI to study encoding of two stimulus categories with different encoding durations. In this study the inferior frontal junction (IFJ), a region involved in the selection and allocation of information into working memory (Courtney et al., 1996), was preferentially implicated in visual working memory encoding, but was also prominent during maintenance. One interpretation of the findings from the current study is that the frontally mediated top-down, selective attention processes observed by others during maintenance (Todd et al., 2011; Gazzaley and Nobre, 2012) do not operate across this entire task component and rather are engaged transiently to enable the transition from information input to maintenance, and from maintenance to memory search and ongoing retrieval.

There are additional considerations which may contribute to the observed overlap of neural activity from working memory encoding to maintenance. To a limited degree, the extension of neural activity from encoding into the maintenance period may be influenced by the averaging procedure used in this study. However, the time frame of the individual frontal amplitude reduction effects which span encoding and early maintenance both exceed that of the 0.77 s averaging window. It has been suggested that aging may be associated with a temporal lag associated with the greater use of reactive strategies (Dew et al., 2012). This could effectively lead to a continuation of encoding-related processes into the maintenance period. In this investigation, there was a greater number of females than males, particularly in the older group. Gender has been shown to influence performance on tasks of spatial, verbal, autobiographical, and emotional memory (Andreano and Cahill, 2009), and activation of brain regions including the primary sensorimotor, premotor cortex, superior parietal and lateral sulcus during visually guided movements (Gorbet and Sergio, 2007). The gender bias of this sample represents a limitation of the current study. Without a pre-midlife comparison it is not clear whether the overlap of neural activity across working memory components was influenced by age of the cohort. Future studies could extend on this work by examining age-related changes in neural activity in large cohorts which encompass the entire adult lifespan.

## Summary and conclusions

In the case of working memory, the neurocognitive aging literature emphasizes task difficulty and capacity limitations as key determinants of brain activation changes in older adults (Reuter-Lorenz and Cappell, 2008; Schneider-Garces et al., 2010). Findings from this study, which utilized the 13 Hz SSVEP, demonstrate that age-associated increases or decreases in neural activation also depend on the specific working memory operation (i.e., encoding, maintenance, retrieval). Importantly, this study suggests that declines to working memory which occur after midlife may be due to under activation of frontal regions during working memory retrieval.

## Conflict of interest statement

This work was financially supported by Swisse Vitamins and Barry Callebaut.
